# MicroRNA-21-3p, a Berberine-Induced miRNA, Directly Down-Regulates Human Methionine Adenosyltransferases 2A and 2B and Inhibits Hepatoma Cell Growth

**DOI:** 10.1371/journal.pone.0075628

**Published:** 2013-09-30

**Authors:** Ting-Fang Lo, Wei-Chung Tsai, Shui-Tein Chen

**Affiliations:** 1 Institute of Biochemical Sciences, College of Life Science, National Taiwan University, Taipei, Taiwan; 2 Institute of Biological Chemistry and Genomics Research Center, Academia Sinica, Taipei, Taiwan; University of Navarra School of Medicine and Center for Applied Medical Research (CIMA), Spain

## Abstract

Methionine adenosyltransferase (MAT) is the cellular enzyme that catalyzes the synthesis of S-adenosylmethionine (SAM), the principal biological methyl donor and a key regulator of hepatocyte proliferation, death and differentiation. Two genes, MAT1A and MAT2A, encode 2 distinct catalytic MAT isoforms. A third gene, MAT2B, encodes a MAT2A regulatory subunit. In hepatocellular carcinoma (HCC), MAT1A downregulation and MAT2A upregulation occur, known as the MAT1A:MAT2A switch. The switch is accompanied with an increasing expression of MAT2B, which results in decreased SAM levels and facilitates cancer cell growth. Berberine, an isoquinoline alkaloid isolated from many medicinal herbs such as *Coptis chinensis*, has a wide range of pharmacological effects including anti-cancer effects. Because drug-induced microRNAs have recently emerged as key regulators in guiding their pharmacological effects, we examined whether microRNA expression is differentially altered by berberine treatment in HCC. In this study, we used microRNA microarrays to find that the expression level of miR-21-3p (previously named miR-21*) increased after berberine treatment in the HepG2 human hepatoma cell line. To predict the putative targets of miR-21-3p, we integrated the gene expression profiles of HepG2 cells after berberine treatment by comparing with a gene list generated from sequence-based microRNA target prediction software. We then confirmed these predictions through transfection of microRNA mimics and a 3′ UTR reporter assay. Our findings provide the first evidence that miR-21-3p directly reduces the expression of MAT2A and MAT2B by targeting their 3′ UTRs. In addition, an overexpression of miR-21-3p increased intracellular SAM contents, which have been proven to be a growth disadvantage for hepatoma cells. The overexpression of miR-21-3p suppresses growth and induces apoptosis in HepG2 cells. Overall, our results demonstrate that miR-21-3p functions as a tumor suppressor by directly targeting both MAT2A and MAT2B, indicating its therapeutic potential in HCC.

## Introduction

Methionine adenosyltransferase (MAT) is the cellular enzyme that catalyzes the synthesis of S-adenosylmethionine (SAM), the principal biological methyl donor and a key regulator of hepatocyte proliferation, death, and differentiation [1], [2]. Two mammalian genes, MAT1A and MAT2A, encode 2 distinct MAT isoforms. A third gene, MAT2B, encodes an MAT2A regulatory subunit. MAT1A is specifically expressed in the adult liver, whereas MAT2A is widely distributed [3]–[5]. Because MAT isoforms differ in catalytic kinetics and regulatory properties, MAT1A-expressing cells have considerably higher SAM levels than do MAT2A-expressing cells [6], [7]. In hepatocellular carcinoma (HCC), the down-regulation of MAT1A gene and the up-regulation of MAT2A occur, known as the MAT1A:MAT2A switch [8]–[11]. The switch results in lower SAM contents, which provide a growth advantage to hepatoma cells [2], [4], [6], [12], [13]. SAM can selectively induce pro-apoptotic Bcl-X_S_ in hepatoma cells, but not in normal hepatocytes, through alternative splicing [14]. In addition, increased MAT2B expression in HCC also results in decreased SAM levels and facilitates cancer cell growth. [15]. Because MAT2A and MAT2B play crucial role in facilitating the growth of hepatoma cells, they are valid targets for antineoplastic therapy. Recent studies have shown that silencing MAT2A and MAT2B by using small interfering RNA substantially suppress growth and induce apoptosis in hepatoma cells [16]–[19].

Berberine, an isoquinoline alkaloid isolated from various medicinal herbs such as *Coptis chinensis*, has a wide range of pharmacological effects including anti-cancer, anti-microbial, anti-inflammatory, and anti-diabetic effects [20]–[23]. Recent studies have focused on its anti-tumor effects, including anti-proliferation, anti-invasion and apoptosis induction in broad tumor cell types [23]–[32]. In HCC, berberine has been reported to inhibit cell growth and survival through cell cycle arrest and the activation of autophagic and mitochondrial apoptotic cell death [33]–[35].

MicroRNAs (miRNAs) are small non-coding RNA molecules composed of 21–23 nucleotides that play a critical role in a wide variety of biological processes, including development, proliferation, and death [36], [37]. The deregulated expression of miRNAs is observed in numerous human cancer types, and they can act as tumor suppressors or oncogenes in the tumorigenic process [38], [39]. Mature miRNAs typically direct their posttranscriptional repression by pairing the seed region of the miRNA to the 3′ UTRs of target genes, leading to mRNA destabilization and translational silencing [40], [41]. The processing of the precursor miRNA (pre-miRNAs) hairpin generates an miRNA duplex, which consists of a guide strand and a passenger strand (also termed miRNA and miRNA*). By convention, a guide strand is selectively loaded onto an Argonaute (AGO) protein to form an miRNA-induced silencing complex (miRISC), and the passenger strand, because of its lower steady-state level, is believed to be preferentially degraded [42]. However, current research shows that numerous miRNA* species accumulate to substantial levels, and that endogenous miRNA genes do not universally exclude miRNA* species from functional miRISC complexes, suggesting that miRNA* species should be considered [43]–[48].

In this study, we used miRNA microarray expression analysis and found that the expression level of miR-21-3p (previously named miR-21*) increased after berberine treatment in the HepG2 human hepatoma cell line. Based on the integration of the gene expression profiles of HepG2 cells after berberine treatment and the gene list that was generated by the sequence-based miRNA target prediction software, we identified MAT2A, DIDO1, EEF2K, NBPF8, and TMWM137 as putative miR-21-3p targets. Subsequent experiments confirmed that miR-21-3p directly suppressed MAT2A expression, as well as the expression of MAT2B by binding to 3′ UTRs. In addition, an overexpression of miR-21-3p raised intracellular SAM contents that have been proven to impair the growth of hepatoma cells. We also found that miR-21-3p reduced the expression of EEF2K, which has been reported as a valid target for anti-cancer treatment because it allows tumor growth and resists cell death. [49]. Consequently, miR-21-3p overexpression suppressed growth and induced apoptosis in HepG2 cells. Our results demonstrate that miR-21-3p functions as a tumor suppressor, indicating its therapeutic potential in HCC.

## Materials and Methods

### Cell Culture and Treatment

The human HepG2 cells and HEK 293T cells were originally obtained from the American Tissue Culture Collection (ATCC, USA). The human HCC HepG2 cell line cultured in Minimum Essential Medium Eagle (Sigma) was supplemented with 10% fetal bovine serum (FBS) (Invitrogen), 2.2 g/L of sodium bicarbonate (Sigma), 0.1 mM of non-essential amino acids (Caisson), 1 mM of sodium pyruvate (Invitrogen), and 10 of ml/L penicillin-streptomycin-amphotericin solution (Biological Industries). Human HEK-293T cells were maintained in high-glucose Dulbecco’s modified eagle medium (Invitrogen) supplemented with 10% FBS and 3.7 g/L of sodium bicarbonate (Sigma). Both cell lines were cultured at 37°C and 5% CO_2_.

A 50-mM stock solution of berberine chloride (Sigma-Aldrich) was prepared in dimethyl sulfoxide (DMSO). Cells were treated with 40-µM of berberine chloride or 0.08% DMSO as the control.

### RNA Isolation

Total RNA was extracted using a TRIZOL reagent (Invitrogen) according to the manufacturer’s protocol. The total RNA quantity was measured using a NanoDrop ND-1000 spectrophotometer (Nanodrop Technologies). The total RNA quality and integrity were assayed using an Agilent 2100 bioanalyzer with an RNA 6000 nano kit (Agilent Technologies).

### Microarray

The miRNA profiling was performed using an Agilent human miRNA Microarray R14 V2 containing 866 human miRNAs. The labeling and hybridization of total RNA were performed by following the standard protocol of Agilent’s miRNA microarray system. Microarrays were scanned following the Agilent microarray scanner protocol, and image analysis and quantitation were performed using the Agilent Feature Extraction software (Agilent Technologies). GeneSpring Gx software was used to identify miRNAs that were differentially expressed (fold-change >2) between the berberine-treated and untreated samples. The gene expression microarray was performed using a HumanHT-12 v4 Expression BeadChip (Illumina). The labeled cRNA was generated from an RT-IVT Kit (Ambion) and a TotalPrep RNA amplification kit (Illumina). The labeled cRNA was then hybridized to microarrays following the manufacturer’s protocol. GenomeStudio software (Illumina) was used to identify miRNAs that were differentially expressed (fold-change >1.5 and *P*<0.05) between the berberine-treated and untreated samples. All microarray data were deposited in the NCBI GEO database (GSE47822).

### Quantitative Real-time RT-PCR (qRT-PCR)

#### MicroRNA assays

Total RNAs (1 ng) were reverse-transcribed into cDNA by using TaqMan Small RNA Assays kits with hsa-miR-21-3p-, hsa-miR-21-5p- or RUN6B-specific RT primers (Invitrogen). The microRNA expression levels were normalized to RNU6B levels.

#### Gene expression assays

Total RNAs (1 µg) were reverse-transcribed into cDNA by using M-MuLV Reverse Transcriptase (Thermo) and Oligo(dT)12–18 primers (Invitrogen) according to the manufacturer’s protocol. The cDNA were then used for a real-time PCR with a LightCycler 480 SYBR Green I Master (Roche) by using MAT1A primers 5′-GCCAAGGGCTTTGACTTC-3′ and 5′-CTGTCTCGTCGGTAGCATA-3′, MAT2A primers 5′-ACAATCTACCACCTACAGCC-3′ and 5′-CCAACGAGCAGCATAAGC-3′, MAT2B primers 5′-TGGTTTCAGAAGAGCAAGAC-3′ and 5′-ATTCCCAGAAGCATCCAC-3′, DIDO1 primers 5′-GATGAGGAGCCTGGAGAC-3′ and 5′-AGAAATGCCCACACAATCG-3′, EEF2K primers 5′-GGCAAACTCCTTCCACTTCA-3′and 5′-CATCATCCAGCCATTCCC-3′, NBPF8 primers 5′-CAGGACATCGGTGGAATCA-3′ and 5′-CTTCTGTAGGGCTGGCAT-3′, TMEM137 primers 5′-GAAGACTGGTTGAGTGGGAT-3′and 5′ TGTCACAGGCAAGTTCACAT 3′, and GAPDH primers 5′- GGTATCGTGGAAGGACTCAT-3′ and 5′-CCTTGCCCACAGCCTTG-3′. The gene expression levels were normalized to GAPDH levels.

### Transfection of microRNA Mimics and Inhibitors

All of the miRNA mimics and inhibitors were purchased from Thermo Scientific Dharmacon. The HepG2 cells were transfected at a density of 5×10^4^ cells per well in a 24-well culture plates with either 50 nM of hsa-miR-21-3p or negative-control mimics, or with either 100 nM of hsa-miR-21-3p or negative-control inhibitors by using the DharmaFECT 4 transfection reagent (Thermo Scientific Dharmacon) according to the manufacturer’s instructions. Cells were incubated for 24 h or 48 h with the microRNA mimics or inhibitors prior to RNA purification for gene expression analysis, and were incubated for 72 h for protein expression analysis.

### Western Blotting

Total cell lysates were prepared using a lysis buffer (7 M of urea, 4% CHAPS, 2 M of thiourea, 40 mM of Tris, 65 mM of dithioerythritol). Protein samples were separated using 12.5% SDS-PAGE and then transferred to PVDF membranes. The following primary antibodies were purchased from GeneTex for used: rabbit polyclone anti-MAT1A (1∶800), anti-MAT2A (1∶1000), anti-MAT2B (1∶1000), and anti-GAPDH (1∶3000). The goat polyclonal anti-rabbit IgG antibody conjugated with HRP (1∶5000, abcam) was used as the secondary antibody. The bands were imaged using the LAS-4000 mini luminescent image analyzer (Fujifilm). The quantification of western blot analysis was achieved by using Image J software. The protein expression levels were normalized to the GAPDH levels.

### Construction of the Luciferase Reporter Plasmids

Full-length 3′ UTRs were amplified from the genomic DNA of HepG2 cells through a PCR. The forward primer with an SpeI restriction site (5′-ATAACTAGTGTGTTAGCCTTTTTTCCCCAG-3′) and the reverse primer with an HindIII restriction site (5′- ATAAAGCTTGCACTTTCTGCTTAGGGCAA-3′) were used to amplify the MAT2A 3′ UTR. The forward primer with an MluI restriction site (5′-ATAACGCGTTGGCACTTTTTAAAGAACAAAGG-3′) and the reverse primer with an HindIII restriction site (5′-ATAAAGCTTAAAAATTAAAGCAACAAAAGAACAA-3′) were used to amplify the MAT2B 3′ UTR. The PCR products were then cloned into the pMIR-REPORT Luciferase miRNA Expression Reporter Vector (Invitrogen), and all inserted sequences in the 3′ UTR constructs were checked using the ABI PRISM DNA sequencer.

### Mutagenesis

The mutagenesis of the target sequence of hsa-miR-21-3p in MAT2A and MAT2B 3′ UTRs was performed using the QuikChange site-directed mutagenesis kit (Agilent) according to the manufacturer’s standard protocol. For the mutagenesis of Site 1 (+180–200) and Site 2 (+1267–1288) in the MAT2A 3′ UTR, 5′-CAGCTCTGCCCTCCCTTCTGTTGATATCAGCCAGACCCC-3′ and 5′-CACTAAATTCATTATAATGGTGAACAAGATATCTAGGGACAGAATAGCAAGCCCAACT-3′ were used. For the mutagenesis of the target site (+399–418) in the MAT2B 3′ UTR, 5′-TTTGATCTGAGCTCAGGCAAAGCAAATAATGGATATCAATGATTTTTATACTATTTCACACAATTTAA-3′ was used. All mutated sequences, including Site 1, Site 2 and the double mutation of the MAT2A and MAT2B 3′ UTR mutant constructs, were checked through DNA sequencing.

### Dual Luciferase Reporter Assay

The HEK-293T and HepG2 cells were seeded at a density of 5×10^4^ cells per well in a 24-well culture plates the day before transfection. The HEK-293T and HepG2 cells were tri-transfected with each pMIR-REPORT-3′ UTR construction (300 ng), the control Renilla luciferase reporter plasmid pRL-TK from Promega (10 ng), and either 50 nM of hsa-miR-21-3p or negative-control mimics by using the Lipofectamine 2000 transfection reagent (Invitrogen) according to the manufacturer’s instructions. Luciferase assays were performed after transfection for 48 h by using the Dual Luciferase reporter assay kit (Promega) and the Paradigm detection platform (Beckman) according to the manufacturer’s protocol. The firefly luciferase activity was normalized relative to the Renilla luciferase activity.

### Measurement of Intracellular SAM Concentration

After 50 nM of hsa-miR-21-3p or the negative-control mimics transfection for 72 h, the HepG2 cells were trypsinized and counted. The intracellular SAM of 5×10^4^ HepG2 cells pellet was resuspended in 30 µL of an extraction solution (0.2% perchloric acid plus 0.08% (v/v) 2-mercaptoethanol in ddH_2_O). Cells were incubated at room temperature for 1 h and vortexed every 5 min. The suspension was centrifuged at 4°C at 10000×g for 5 min, and then the supernatant was collected. SAM levels in the supernatants were quantified using the Bridge-It SAM fluorescence assay kit (Mediomics) and detected using SpectraMax plate reader (Molecular Devices) according to the manufacturer’s instructions.

### Cell Proliferation Assay

The HepG2 cells were seeded at a density of 1×10^4^ cells per well in a 96-well culture plates the day before transfection. After 50 nM of hsa-miR-21-3p or the negative-control mimics were transfected for 24 h, the cultured media were refreshed with complete media containing the BrdU reagent, and incubated for an additional 24 hour for BrdU incorporation. The BrdU incorporation was quantified by using the BrdU cell proliferation colorimetric ELISA kit (abcam) according to the manufacturer’s protocol and detected using the MRX II microplate reader (DYNEX).

### Detection of Apoptosis by Using Flow Cytometry

After 50 nM of hsa-miR-21-3p or the negative-control mimics were transfected for 72 h, the HepG2 cells were trypsinized and counted. Apoptosis was detected by measuring the sub-G1 population by using flow cytometry with propidium iodide (PI) staining. In brief, the cells were fixed in 70% ethanol on ice for 15 min, and stained with the PI staining solution (20 µg/mL PI, 0.1% Triton-X 100, and 0.2 mg/mL RNase A in PBS) for 30 min at room temperature and analyzed using CyAn ADP (Beckman Coulter) flow cytometry with Summit software.

## Results

### Increased miR-21-3p Expression After Berberine Treatment in HepG2 Cell Lines

**Figure 1:**
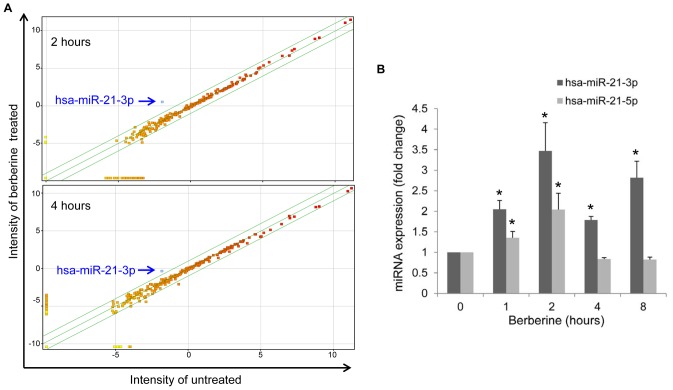
Berberine treatment increases the expression of hsa-miR-21-3p in HepG2 cell lines. (A) Scatter plot showing a comparison of the miRNA expression profiles between berberine-treated and untreated samples. The blue arrow indicates that hsa-miR-21-3p is the only miRNA that increased in the HepG2 cell line after 40-µM berberine treatment for 2h and 4 h. The green lines represent the 2 and 0.5 boundary values for fold induction. (B) HepG2 cells stimulated by the time course of 40-µM berberine treatment for up to 8 h compared with the untreated control. The hsa-miR-21-3p and hsa-miR-21-5p levels were then measured using qRT-PCR and expressed as fold expression. Data are presented as the mean ± standard error of 3 independent experiments. “*” indicates a significant difference with P<0.05.

Because xenobiotic drug-induced miRNAs have recently emerged as key regulators in guiding their pharmacological effects and toxicity [50], [51], we examined whether miRNA expression is differentially altered by berberine treatment in HCC. To identify miRNAs induced by berberine treatment, miRNA profiling was performed with an Agilent human miRNA microarray containing probes for 866 human miRNAs. Comparing the miRNA profiles of 40-µM of the berberine-treated HepG2 human hepatoma cell line to those of control cells sampled after 2 h and 4 h of treatment shows that only hsa-miR-21-3p (previously named miR-21*) had increased in the HepG2 cell line after berberine treatment (4-fold increase) (Fig. 1A). To further assess the relevance of miR-21-3p in berberine treatment, real-time PCR assays were used to measure the miR-21-3p expression in HepG2 stimulated by the time course of berberine treatment for up to 8 h compared with the untreated control. As shown in Fig. 1B, miR-21-3p levels started to increase substantially by 1 h (2.1-fold increase) after treatment, peaked at 2 h (3.5-fold increase), and persisted for 8 h (2.8-fold increase). In addition, miR-21-5p (previously named miR-21) increased 2-fold after 2 h of treatment, whereas no significant differences in miR-21-5p expression were detected at 4 h and 8 h. These results show that berberine treatment could significantly up-regulate miR-21-3p expression.

### Multiple Species Alignments Show that miR-21-3p is Conserved Over the Mammalian Evolution

**Figure 2:**
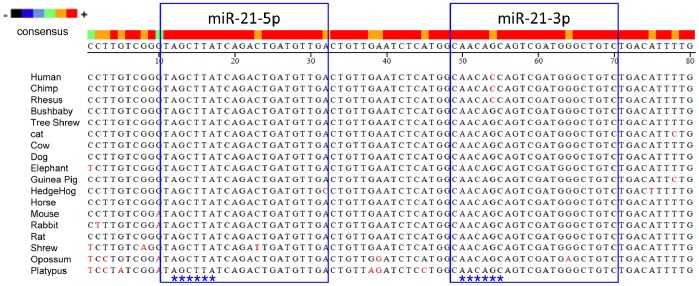
MIR21 is conserved over the mammalian evolution. The MIR21 gene sequences of 18 mammalian species were obtained from the UCSC genome browser. Multiple species alignment was performed using the ClustalV Method within MegAlign software. The asterisk mark indicates the seed region of miRNAs.

The final fate of the miRNA* strand, either expressed abundantly as a potential functional guide miRNA or degraded to a passenger strand, may be destined across evolution [48]. Well-conserved miRNA* strands in seed sequences may afford potential opportunities for contributing to the regulation network [46]. As shown in Fig. 2, we analyzed the *MIR21* gene with respect to the 18-way alignments of mammalian genomes from the UCSC genome browser, and found that miR-21-3p shows conservation over the mammalian evolution. Based on the results of sequence comparisons, we found that human *MIR21* has the closest evolutionary relationships with chimpanzee and rhesus macaque *MIR21*. Furthermore, one nucleotide substitution is in the seed region of miR-21-3p from humans, chimpanzees, and rhesus macaques compared to the remaining 15 mammals. This one nucleotide substitution, *MIR21* (+54 G to C), replaces the fifth nucleotide of the conserved seed region of miR-21-3p, which may alter the regulatory role of miR-21-3p in humans, chimpanzees and rhesus macaques from remaining 15 mammals.

### MicroRNA-21-3p Target Prediction and Validation

**Figure 3:**
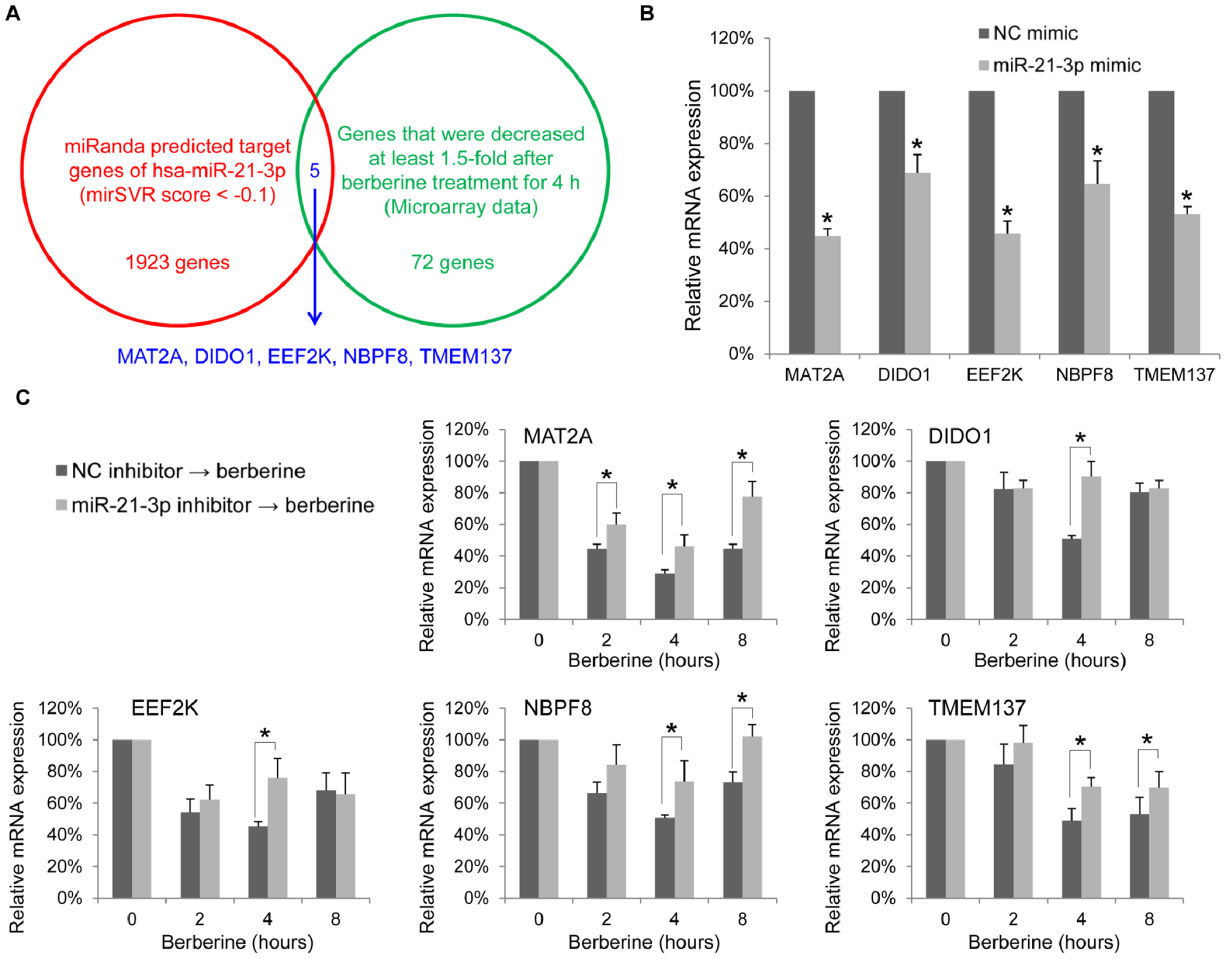
MAT2A, DIDO1, EEF2K, NBPF8, and TMEM137 are regulated by berberine-induced miR-21-3p. (A) The mRNA targets of miR-21-3p were predicted using the miRanda algorithm and an mirSVR score threshold of −0.1. Predicted genes were overlapped with the microarray data of negatively regulated genes after berberine treatment for 4 h in HepG2 cells, thereby identifying 5 gene targets as indicated. (B) The relative mRNA expression levels of the predicted target genes in HepG2 cells were measured using qRT-PCR by comparing the transfection of 50 nM of miR-21-3p mimics and negative-control (NC) mimics. (C) After transfection of 100 nM of miR-21-3p hairpin inhibitors or negative-control (NC) inhibitors into the HepG2 cells for 24 h, the cells were stimulated by the time course of 40-µM berberine treatment for up to 8 h compared with the untreated control. The relative mRNA expression levels of the predicted target genes were measured using a qRT-PCR. Data are presented as the mean ± standard error of 3 independent experiments. “*” indicates a significant difference with P<0.05.

Identifying functionally important target genes of specific miRNA and understanding the mechanisms of their actions are essential to uncovering its biological function [52]. To predict the putative targets of miR-21-3p, we integrated the gene expression profiles of HepG2 cells after berberine treatment and compared them with the gene list that we generated from sequence-based miRNA target prediction software. As shown in Fig. 3A, the mRNA targets of miR-21-3p were predicted using the miRanda algorithm and an mirSVR score threshold of -0.1 [53]. The predicted genes were overlapped with the microarray data of negatively regulated genes (more than a 1.5-fold decrease and *P*<0.05) after 40-µM berberine treatment for 4 h in HepG2 cells. Five gene targets, including MAT2A (methionine adenosyltransferase II, alpha), DIDO1 (death inducer-obliterator 1), EEF2K (eukaryotic elongation factor-2 kinase), NBPF8 (predicted: *Homo sapiens* neuroblastoma breakpoint family, member 8), and TMEM137 (predicted: *Homo sapiens* transmembrane protein 137) were identified using this prediction strategy. We next confirmed these predictions by performing gain-of-function and loss-of-function experiments with miRNA mimics and inhibitors, and confirmed whether the miR-21-3p inhibitor could successfully rescue the berberine function in lowering the expression levels of selected targets. Figure 3B shows that transfecting 50 nM of miR-21-3p mimics into HepG2 cells for 24 hours resulted in a >50% decrease in the mRNA expression of MAT2A and EEF2K, and a <50% decrease in the mRNA expression of DIDO1, EEF2K, and NBPF8. After the transfection of 100 nM of miR-21-3p hairpin inhibitors or negative-control inhibitors into HepG2 cells for 24 h, the cells were stimulated by the time course of 40-µM berberine treatment for up to 8 h compared with the untreated control. Figure 3C shows that miR-21-3p inhibitor could successfully rescue the berberine function in the lowering mRNA expression levels of predicted targets. These results suggest that MAT1A, DIDO1, EEF2K, NBPF8, and TMEM137 were the targets of miR-21-3p. We then focused on methionine adenosyltransferase (MAT) which is strongly associated with hepatocellular carcinoma.

### MicroRNA-21-3p Reduces the Expression of Methionine Adenosyltransferases 2A and 2B

**Figure 4:**
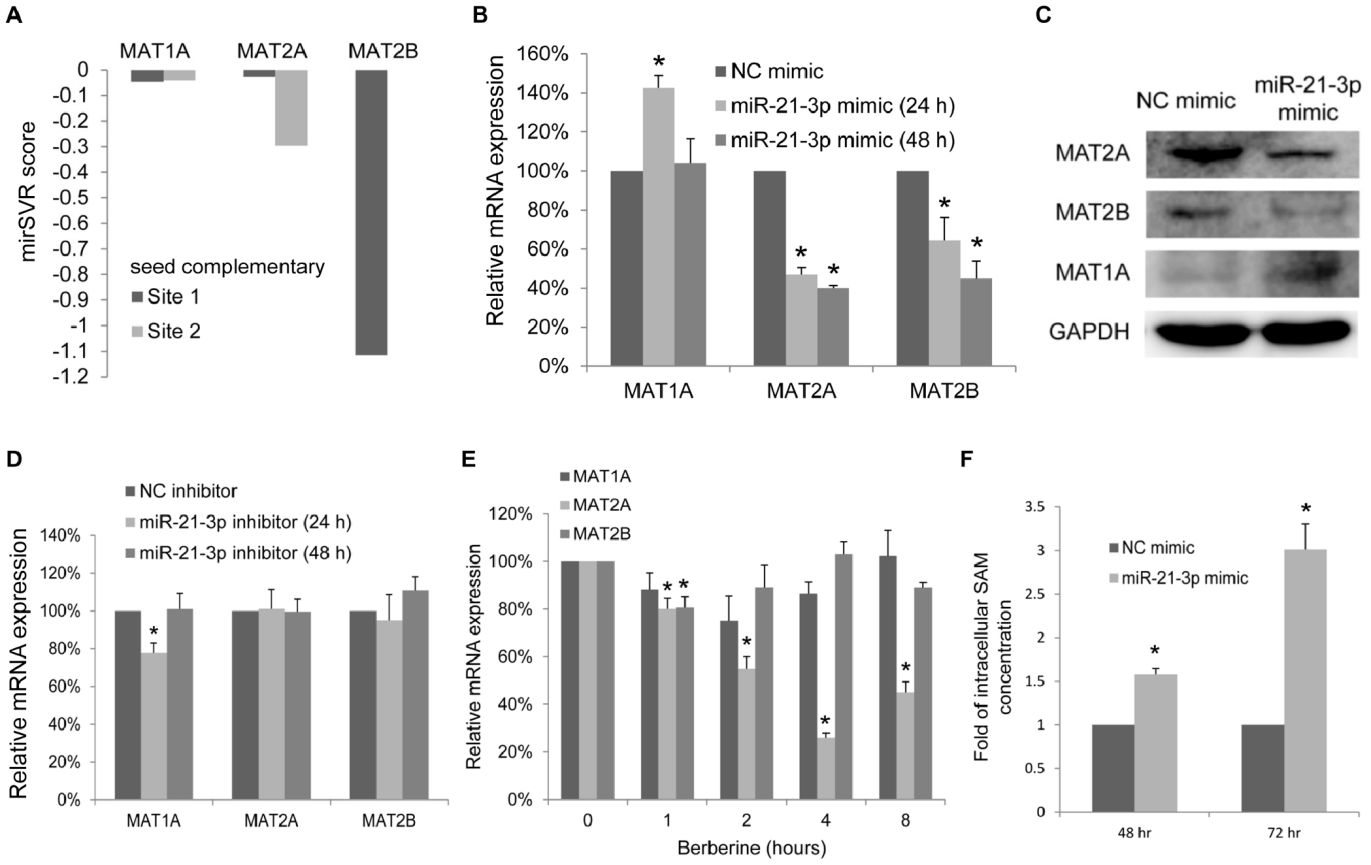
MicroRNA-21-3p and berberine induce differential changes in MAT expression and miR-21-3p up-regulates SAM contents. (A) The histogram shows the mirSVR score of each seed complementary site in the 3′ UTRs of MAT1A, MAT2A, and MAT2B. (B) The relative mRNA expression levels of MAT1A, MAT2A, and MAT2B in HepG2 cells were measured using a qRT-PCR by comparing the transfection of 50 nM of miR-21-3p mimics and negative-control (NC) mimics for 24 h and 48 h. Data are presented as the mean ± standard error of 3 independent experiments. (C) Western blot analysis showing the protein levels of MAT2A, MAT2B and MAT1A after the transfection of 50 nM of miR-21-3p mimics or negative-control mimics for 72 h. GAPDH was used as the loading control. (D) The relative mRNA expression levels of MAT1A, MAT2A, and MAT2B in HepG2 cells were measured using a qRT-PCR by comparing the transfection of 100 nM of miR-21-3p inhibitors and negative-control inhibitors for 24 h and 48 h. Data are presented as the mean ± standard error of 3 independent experiments. (E) The HepG2 cells stimulated by the time course of 40-µM berberine treatment for up to 8 h compared with the untreated control. The relative mRNA expression levels of MAT1A, MAT2A, and MAT2B were measured using a qRT-PCR. (F) After 50 nM of miR-21-3p mimics or negative-control mimics transfection for 72 h, the intracellular SAM concentration of the HepG2 cells was quantified. The SAM levels were expressed as a fold expression. Data are presented as the mean ± standard error of 3 independent experiments. “*” indicates a significant difference with *P*<0.05.

After determining that MAT2A is the putative target of miR-21-3p, we investigated whether the expression levels of MAT family members including MAT1A, MAT2A, and MAT2B, could be altered by miR-21-3p. The histogram in Fig. 4A shows the miRanda-mirSVR scores of each seed complementary site in the 3′ UTR of MAT1A, MAT2A, and MAT2B. We found that MAT2A as well as MAT2B scored lower than -0.1, suggesting MAT2B might also be a target of miR-21-3p. To demonstrate the relationship between miR-21-3p and the expression levels of the MAT family, miR-21-3p mimics or negative-control mimics were transfected into HepG2 cells. The mRNA and protein expression levels of the MAT family members were subsequently assayed using real-time quantitative PCR and western blotting. Figure 4B shows that transfecting 50 nM of miR-21-3p mimics into HepG2 cells for 48 h resulted in a >50% decrease in the mRNA expression of MAT2A and MAT2B, but not in that of MAT1A. Furthermore, after the transfection of hsa-miR-21-3p mimics into HepG2 cells for 72 h, the protein expression levels of MAT2A and MAT2B showed a 2.6-fold and a 3.4-fold decrease, respectively. Furthermore, the MAT1A protein expression levels showed a 1.7-fold increase (Fig. 4C). By contrast, for the loss-of function experiments, the hairpin inhibitors of miR-21-3p were transfected into HepG2 cells to inhibit functions of endogenous miR-21-3p with negative control inhibitors as control. As shown in Fig. 4D, the MAT1A was 1.3-fold decreased by miR-21-3p inhibitors after transfection for 24 h, but not for 48 h. The expression levels of MAT2A and MAT2B remained unchanged after transfection for 24 h and 48 h. The MAT2B expression levels did not change notably according to our microarray data shown in Fig. 3A. To assess the relevance of berberine treatment and the expression levels of the MAT family members, real-time PCR assays were used to measure mRNA expression in HepG2 cells stimulated by the time course of berberine treatment for up to 8 h compared with the untreated control (0.08% DMSO). As shown in Fig. 4E, MAT2A levels started to decreasing substantially by 2 h (1.8-fold decrease) after treatment, reached the lowest value at 4 h (3.8-fold decrease), and remained constant for 8 (2.2-fold increase). The decreased levels of MAT2A were associated with higher miR-21-3p levels after berberine treatment for 2 h, 4 h, and 8 h, and the comparisons are shown in Fig. 1B. However, MAT1A and MAT2B expression levels did not change substantially which was consistent with our microarray data. Overall, our findings are the first evidence indicating that berberine treatment reduced MAT2A, and that the overexpression of miR-21-3p reduced the expression of both MAT2A and MAT2B.

### MicroRNA-21-3p up-regulates Intracellular SAM Contents in Hepatoma Cells

The increased expression of MAT2A and MAT2B in HCC results in decreasing SAM levels and facilitates cancer cell growth [6], [7], [15]. After determining that MAT2A and MAT2B are repressed by miR-21-3p, we analyzed the intracellular SAM contents in the HepG2 cell after transfection with miR-21-3p mimics and negative-control mimics as the control for 48 h and 72 h. As shown in Fig. 4F, the intracellular SAM contents were 1.6-fold and 3.0-fold increased by miR-21-3p mimics after transfection for 48 h and 72 h. These results indicate that the overexpression of miR-21-3p raised intracellular SAM contents, which have been proven to impair the growth of hepatoma cells.

### MAT2A and MAT2B are Direct Targets of miR-21-3p

**Figure 5:**
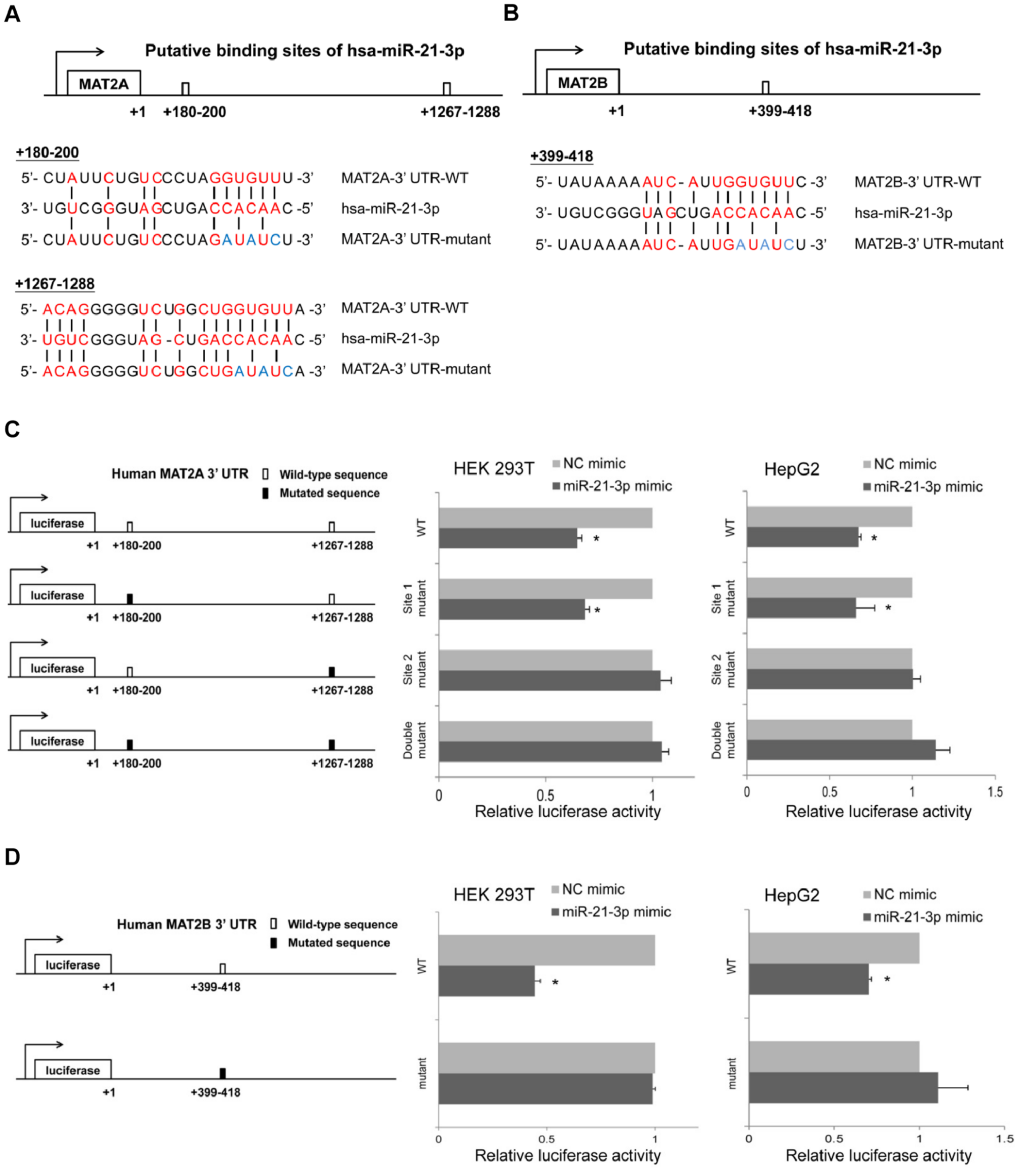
A dual luciferase reporter assay shows that MAT2A and MAT2B are direct targets of miR-21-3p. (A and B) Top: A schematic representation of the putative 3′ UTR binding sites of MAT1A or MAT2B for miR-21-3p. Bottom: The seed binding sites in 3′ UTR were mutated. (C and D) Left: The MAT2A or MAT2B 3′ UTR-wild-type or 3′ UTR-mutant was inserted downstream of the luciferase of the pMIR-reporter vector. Middle: The relative luciferase activity of cells was measured on HEK293T cells. Right: The relative luciferase activity of cells was measured on HepG2 cells. A total of 5×10^4^ cells were tri-transfected with each wild-type-UTR-reporter or mutant-UTR-reporter constructs (300 ng), the control Renilla luciferase reporter plasmid pRL-TK from Promega (10 ng), and either 50 nM of hsa-miR-21-3p or negative-control mimics. Data are presented as the mean ± standard deviation of 3 independent experiments. “*” indicates a significant difference with *P*<0.05.

Because MAT2A and MAT2B decreased after miR-21-3p mimics transfection, we investigated the relevance of miR-21-3p and the 3′ UTRs of MAT2A and MAT2B. Full-length wild-type or mutant 3′ UTRs of MAT2A and MAT2B were separately cloned into luciferase reporter vectors (Figs. 5A and 5B, respectively), and the dual luciferase reporter assay system was used to quantitate the reporter activity. Fig. 5C shows that miR-21-3p suppressed the expression (a 1.6-fold decrease in HEK 293T cells and a 1.5-fold decrease in HepG2 cells) of the luciferase reporter containing the MAT2A 3′ UTR, suggesting that miR-21-3p directly regulates the MAT2A 3′ UTR. To confirm whether the miR-21-3p cleaves the MAT2A 3′ UTR through miRNA:mRNA seed pairing, the site-directed mutagenesis of the putative miR-21-3p binding sequence on the MAT2A 3′ UTR was performed. By using MicroRNA.org (http://www.microrna.org), a comprehensive resource of microRNA target predictions, miR-21-3p was predicted to target 2 sites (Site 1: +180–200 and Site 2: +1267–1288) in the 3′ UTR of MAT2A. The results show that miR-21-3p suppressed the expression (a 1.5-fold decrease in both HEK 293T cells and HepG2 cells) of the luciferase reporter containing Site 1 mutated MAT2A 3′ UTR. Conversely, the expression levels of the luciferase reporter containing Site 2 mutated MAT2A 3′ UTR did not notably change, similar to the reporter containing the double-mutated (both Site 1 and Site 2) MAT2A 3′ UTR. This Suggests that Site 2 (+1267–1288) in the MAT2A 3′ UTR is the major cleavage site of miR-21-3p (Fig. 5C). In addition, miR-21-3p was predicted to target one seed match (+399–418) in the 3′ UTR of MAT2B. As shown in Fig. 5D, miR-21-3p suppressed the expression (a 2.3-fold decrease in HEK 293T cells and a 1.4-fold decrease in HepG2 cells) of the luciferase reporter containing the MAT2B 3′ UTR, but the expression levels of the luciferase reporter containing the mutated MAT2B 3′ UTR remained unchanged in both HEK 293T cells and HepG2 cells. This Suggests that the seed match (+399–418) in the MAT2B 3′ UTR is the major cleavage site of miR-21-3p. These results showed that MAT2A and MAT2B are both direct targets of miR-21-3p.

### MicroRNA-21-3p Suppresses Growth and Induces Apoptosis in Hepatoma Cells

**Figure 6:**
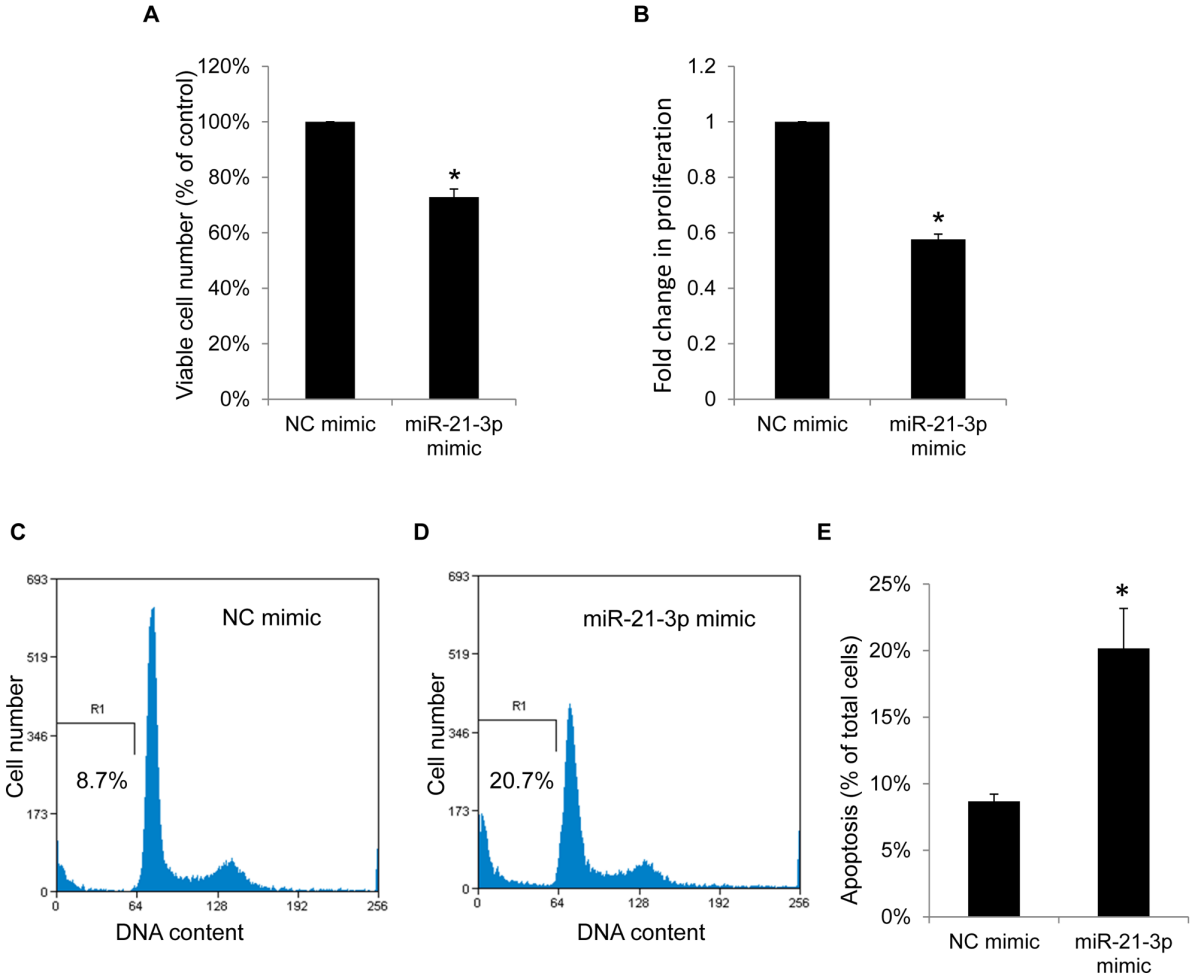
MicroRNA-21-3p suppresses growth and induces apoptosis in HepG2 cells. (A) After 50 nM of miR-21-3p mimic or negative-control mimics were transfected for 48 h, viable HepG2 cells were quantified using a Trypan blue dye exclusion assay. (B) After transfecting 50 nM of miR-21-3p mimics or negative-control mimics into HepG2 cells for 24 h and incubating for an additional 24 h for BrdU incorporation, the cellular proliferation was measured using the BrdU incorporation assay kit and expressed as fold change. (C, D and E) After 50 nM of miR-21-3p mimics or negative-control mimics were transfected for 72 h, apoptosis was detected by measuring the sub-G1 population using flow cytometry with propidium iodide staining. Data are presented as the mean ± standard deviation of 3 independent experiments. “*” indicates a significant difference with *P*<0.05.

To investigate the potential effects of miR-21-3p on cell growth and viability, we measured cell proliferation and viability by using the BrdU incorporation assay and the Trypan blue dye exclusion assay. The results shown in Fig. 6A indicate that miR-21-3p mimics reduced the viable cell numbers in HepG2 cells after transfection for 48 h. In addition, transfecting miR-21-3p mimics into HepG2 cells for 24 h and incubation for an additional 24 h for BrdU incorporation led to the inhibition of cellular proliferation (1.7-fold), compared with transfection with negative-control mimics (Fig. 6B). The effects of miR-21-3p on apoptosis and the cell cycle were evaluated using flow cytometry analysis. After HepG2 cells transfection with miR-21-3p and negative-control mimics as the control for 72 h, the sub-G1 populations of apoptotic cells were quantified. As shown in Figs. 6C, 6D, and 6E, miR-21-3p induced apoptosis in HepG2 cells.

## Discussion

Because drug-induced miRNAs have recently emerged as key regulators in guiding their pharmacological effects and toxicity [50], [51], we examined whether miRNA expression was differentially altered through berberine treatment in HCC. Although the up-regulation of microRNAs in MHCC97-L cells after treatment with the *Coptidis rhizoma* aqueous extract [54] and decreased levels of miR-21-5p in multiple myeloma cells after berberine treatment have been reported [55], we present the first evidence that the expression levels of miR-21-3p (previously named miR-21*) increased after berberine treatment in the HepG2 human hepatoma cell line. Based on multiple species alignments of the 18 currently available mammalian genomes, we found that one nucleotide substitution, MIR21 (+54 G to C), replaced the fifth nucleotide of the conserved seed region of miR-21-3p. This nucleotide substitution may alter the regulatory role of miR-21-3p in humans, chimpanzees and rhesus macaques from remaining 15 mammals. To assist miRNA-based drug development, identifying functionally important target genes of a specific miRNA and understanding the mechanisms of their actions are essential to uncovering biological functions of a specific miRNA [52]. However, the functions of miR-21-3p are currently unknown. We integrated the gene expression profiles of HepG2 cells after berberine treatment and compared them with a gene list generated from sequence-based miRNA target prediction software. We found that MAT1A, DIDO1, EEF2K, NBPF8, and TMEM137 were putative targets. The DIDO1, which has recently been reported to promote BMP-induced progression and apoptosis resistance in melanoma cells [56]. We also found that the transfection of miR-21-3p resulted in a >50% decrease in the mRNA expression of EEF2K, which is a valid target for anti-cancer treatment. Unlike MAT2A and MAT2B, which are strongly related to HCC, EEF2K is increased in many cancers, including HCC, allowing tumor growth and resisting cell death [49]. We then focused on methionine adenosyltransferase (MAT) which is strongly associated with HCC.

HCC is one of the most frequently occurring and deadliest cancers types worldwide [57]. A strong relationship between HCC and MAT has been reported. As mentioned in the literature, a switch in gene expression from MAT1A to MAT2A, known as the MAT1A:MAT2A switch, has been found in liver cancer [8]–[11]. The MAT1A:MAT2A switch, which was accompanied with an increasing expression of MAT2B, results in decreasing SAM levels and facilitates cancer cell growth [2], [4], [6], [12], [13], [15]. Because MAT2A and MAT2B play vital role in facilitating the growth of hepatoma cells, they are valid targets for antineoplastic therapy. During proliferation of liver cells or in HCC, transcription of the MAT2A gene is up-regulated by several transcription factors, including E2F, Sp1, and c-Myb [58], [59]. In addition, histone H4 acetylation and prevalent CpG hypomethylation in MAT2A promoter were found, and the MAT2A mRNA stability was further increased by associated with HuR (Huantigen R) [8]. Besides, the study has shown that SP3 can bind to gene MAT2B promoter and activates MAT2B expression [60]. Potential regulatory miRNAs of MAT1A, which were up-regulated in HCC, were recently reported. Although MAT2A and MAT2B have been predicted to be putative targets of some microRNAs [61]–[63], no study has experimentally determined the direct relationship between miRNA and MAT2A/MAT2B. Our findings provide the first evidence that miR-21-3p directly reduces the expression of MAT2A and MAT2B by directly targeting their 3′ UTRs. In addition, the transfection of miR-21-3p mimics increased intracellular SAM contents. Conflicting results have been reported on intracellular SAM contents increasing or decreasing after the knockdown of MAT2A in hepatoma cells [16]–[18], whereas, intracellular SAM contents were increased in all relevant studies after knockdown of MAT2B or both MAT2A and MAT2B [17]–[19]. Consistent with previous studies, our results showed that miR-21-3p directly reduced the expression of both MAT2A and MAT2B resulting in increasing intracellular SAM contents that have been proven to impair the growth of hepatoma cells. For the loss-of function experiments, the hairpin inhibitors of miR-21-3p were transfected into hepatocytes to inhibit functions of endogenous miR-21-3p with negative control inhibitor as control. However, due to the endogenous low-level expression of passenger strand, the targets of miR-21-3p might not be altered in loss-of function experiments, as shown in Fig. 4D. For this reason, we designed another experiment for combining loss-of function experiments and berberine treatment. As shown in Fig. 3C, miR-21-3p inhibitor could successfully rescue the berberine function in the lowering mRNA expression levels of predicted targets. Additionally, the MAT1A was 1.3-fold decreased after transfection of miR-21-3p inhibitors for 24 h, suggesting that MAT1A was regulated by endogenous miR-21-3p (Fig. 4D). After overexpression of miR-21-3p (Fig. 4B), the mRNA expression level of MAT1A was induced 1.4 fold at 24 h, but mRNA expression level of MAT1A was no change at 48 h. Liu Q et al. have been shown that silencing MAT2A led to the induction of MAT1A expression through increasing intracellular SAM levels, but not through decreasing MAT2A expression [16]. We proposed that up-regulation of the MAT1A protein level after transfection miR-21-3p for 72 h might due to substantially increased SAM levels at 72 h. The induction of MAT1A further up-regulated SAM levels and down-regulated MATII enzyme activities. Therefore, miR-21-3p transfection suppresses growth and induces apoptosis in HepG2 cells.

Further research should be undertaken in the following areas. First, we found that the MAT2B expression levels did not notably change within 8 h of berberine treatment but decreased after miR-21-3p transfection by comparing Fig. 4B with 4E. These results suggested that berberine may regulate MAT2B expression through multiple pathways. The pathways that are inducted by berberine in regulating MAT expression require further investigation. Second, the reason that caused the berberine to enhance the expression of miR-21-3p should be investigated in further studies. Fig. 1 shows that the expression of miR-21-3p increased within 8 hours following berberine treatment. In comparison, the miR-21-5p increased only slightly within the first 2 hours, and remained unchanged up to 8 hours. This suggests that berberine influenced the transcription of MIR21 within first 2 hours, and subsequently influenced the strand selection of the mir21-mir21* duplex products. The enhanced expression of a passenger strand involved a complex process of the microRNA strand selection that has remained ambiguous until now. In the current study, we found that the miR-21-3p, a passenger strand generated from MIR21, was induced after berberine treatment in HepG2 cells. This provides a cell model with information for investigating the microRNA strand selection in the future.

Overall, we found that the expression levels of miR-21-3p increased after berberine treatment in the HepG2 human hepatoma cell line. Our findings are the first evidence that miR-21-3p directly reduces the expression of MAT2A and MAT2B by directly targeting their 3′ UTRs. Furthermore, miR-21-3p overexpression increased intracellular SAM contents, and suppressed growth and induced apoptosis in HepG2 cells. Thus, our results indicate that miR-21-3p functions as a tumor suppressor, suggesting its therapeutic potential in HCC.
